# One-Year Prognosis for Patients Diagnosed with Acute Coronary Syndrome Compared to Those with Chronic Coronary Syndrome Following Complex Coronary Procedures

**DOI:** 10.3390/jcm14030730

**Published:** 2025-01-23

**Authors:** Patrycja Woźniak, Konrad Stępień, Wojciech Wańha, Anna Smukowska-Gorynia, Aleksander Araszkiewicz, Maciej Lesiak, Weronika Jędraszak, Tatiana Mularek-Kubzdela, Sylwia Iwańczyk

**Affiliations:** 1 1st Department of Cardiology, Poznan University of Medical Sciences, 60-355 Poznan, Poland; anna.smukowska-gorynia@usk.poznan.pl (A.S.-G.); aleksander.araszkiewicz@usk.poznan.pl (A.A.); maciej.lesiak@usk.poznan.pl (M.L.); tatiana.mularek-kubzdela@usk.poznan.pl (T.M.-K.); syl.iwanczyk@gmail.com (S.I.); 2 Department of Coronary Artery Disease and Heart Failure, St. John Paul II Hospital, 31-202 Kraków, Poland; konste@interia.eu; 3 Department of Thromboembolic Disorders, Institute of Cardiology, Jagiellonian University Medical College, 31-202 Kraków, Poland; 4 Department of Cardiology and Structural Heart Diseases, Medical University of Silesia, 40-635 Katowice, Poland; wojciech.wanha@gmail.com; 5 National Medical Institute of the Ministry of Interior and Administration, 02-507 Warszawa, Poland; 6 Poznan University of Medical Sciences, 60-355 Poznan, Poland; weronikajedraszak@gmail.com

**Keywords:** acute coronary syndrome, chronic coronary syndrome, coronary artery disease, complex percutaneous coronary intervention

## Abstract

**Background:** Acute coronary syndrome (ACS) remains the primary cause of mortality worldwide. Performing complex coronary intervention in patients with ACS is considered a significant factor for worsening prognosis. This study aimed to evaluate the prognosis of patients with ACS treated with complex procedures compared to patients with chronic coronary syndrome (CCS). **Methods:** Among 980 patients from the Polish Complex Registry, we enrolled 829 consecutive patients who underwent complex percutaneous coronary intervention (PCI) for acute or chronic coronary syndrome with a completed one-year follow-up. The primary endpoint is defined as the major adverse cardiac event (MACE) at 12 months, a composite endpoint including all-cause death, target lesion revascularization, target vessel revascularization, and non-fatal myocardial infarction. **Results:** The incidence of the composite endpoint of MACE at one-year follow-up was comparable between the patients with acute and chronic coronary syndrome who underwent complex PCI (12.4% vs. 7.6%, LogRank *p* = 0.035). Cox multivariate analysis indicated that ACS is an independent risk factor for death at one-year follow-up. Additionally, age and comorbidities, such as heart failure and chronic kidney disease, along with procedural factors, including lesion length and pre-procedural diameter stenosis, are independent predictors of death in patients with complex lesions. Independent risk factors for MACE at one-year follow-up include age, heart failure, previous PCI, in-stent restenosis, and pre-procedural diameter stenosis. **Conclusions:** The prognosis of patients with acute and chronic coronary syndrome in the annual follow-up is comparable in the context of cardiovascular events. The clinical presentation of ACS is an independent risk factor for all-cause death.

## 1. Introduction

Acute coronary syndrome (ACS) continues to be the leading cause of mortality worldwide despite significant advancements in its diagnosis and management, including both pharmacological therapies and coronary interventions [1,2]. The increasing prevalence of complex calcific and diffuse lesions among patients undergoing primary percutaneous coronary intervention (pPCI) serves as a critical prognostic factor. Although effective lesion preparation devices have been developed, independent predictors of stent thrombosis (ST) and in-stent restenosis (ISR), such as total stent length and stent overlap, persist [3]. Moreover, in the ACS patient population, optimizing complex procedures guided by intracoronary imaging and applying advanced calcification modification techniques remain notably infrequent, which may impact long-term prognosis.

Data regarding treatment outcomes for ACS patients undergoing complex interventions are limited. Our objective was to evaluate the prognosis of patients with ACS and compare it to those with chronic coronary syndrome (CCS) in the context of complex coronary procedures.

## 2. Methods

### 2.1. Study Design and Patient Population

The current analysis included all patients from the Polish COMPLEX Registry who completed at least one year of follow-up. The registry is a retrospective, investigator-initiated, high-volume Polish single-center clinical registry comprising 980 consecutive patients with complex coronary artery lesions. These patients presented with ACS or CCS and were treated with new-generation drug-eluting stents (DESs) between September 2015 and December 2021. The detailed inclusion criteria were described by Iwańczyk et al. in their research [3].

The procedure was classified as a complex procedure if it met at least one of the following conditions: lesion length > 40 mm, chronic total occlusion (CTO), multivessel PCI during the same intervention, severe calcification assessed by angiography or intravascular imaging examination, or true bifurcation defined as any lesion involving both the main vessel, proximal or distal and the ostium of the side branch (Medina 1,1,1; 1,0,1; or 0,1,1).

Our analysis excluded patients with left main stem PCI treated during index procedure, patients after cardiogenic shock, thrombolysis before PCI, or patients in whom a 12-month follow-up was unavailable.

All patients were treated with the current generation DES. Specifically, 52% of the patients received sirolimus-eluting stents, primarily Orsiro (BIOTRONIK SE & Co., Berlin, Germany) and Ultimaster (Terumo Corporation, Tokyo, Japan). Everolimus-eluting stents, such as Xience (Abbott Vascular, Santa Clara, CA, USA) and Synergy (Boston Scientific, Marlborough, MA, USA), were used in 44% of the patients, while 4% received zotarolimus-eluting stents (Resolute Onyx, Medtronic, Santa Rosa, CA, USA).

After the index procedure, each enrolled patient underwent clinical follow-up through telephone interviews every six months and during outpatient clinic visits. The ethics committee approved this study.

The patients were divided into ACS and CCS groups based on clinical presentation at admission to compare peri-procedural and one-year outcomes.

### 2.2. Procedure Description

All patients underwent pretreatment with acetylsalicylic acid and P2Y12 inhibitor (clopidogrel, prasugrel, or ticagrelor) as loading doses, followed by intravenous injection of 100 U/kg unfractionated heparin. Prasugrel or ticagrelor was preferred in patients with ACS instead of clopidogrel. We used the bailout glycoprotein IIb/IIIa receptor inhibitor strategy in patients with high thrombus burden.

PCI was performed according to the guidelines and local recommendations [4]. The peri-procedural use of intracoronary imaging and the duration of the prescribed dual antiplatelet therapy (DAPT) depended on the interventional cardiologist’s discretion.

All angiographic data were assessed by Quantitative Coronary Angiography (QCA) by two independent and experienced operators. QAngio XA computer software (QAngio XA User Manual 8.1.2.4, Medis, Leiden, The Netherlands) was used for the QCA analysis.

### 2.3. Endpoint Definitions

The primary endpoint was a major adverse cardiac event (MACE) at 12 months, a composite endpoint including all-cause death, target lesion revascularization (TLR), target vessel revascularization (TVR), and non-fatal myocardial infarction (MI). Secondary endpoints were the single components of MACE, cardiovascular death, ST, and angiographic success as a safety endpoint. TLR was defined as repeated PCI or coronary artery bypass grafting (CABG) in the target segment, including 5 mm proximal and distal to the previously treated lesion. MI was diagnosed according to the Fourth Universal Definition of Myocardial Infarction [5]. Angiographic success was defined as successful stent delivery with a residual diameter stenosis value of 20% without procedural complications. Procedural success was defined as angiographic success in the absence of in-hospital major events, including death, MI, recurrent chest pain requiring TVR or TLR with PCI or CABG, cardiac tamponade requiring pericardiocentesis or surgery, vascular access site complications, stroke, and contrast-induced nephropathy [6].

### 2.4. Statistical Analysis

All continuous variables were presented as means (standard deviation) for a normal distribution or medians (upper and lower quartile) for a non-normal distribution. The normality of the distribution of variables was tested using the Kolmogorov–Smirnov test. Categorical variables were presented as counts and percentages or frequencies. The significance of differences between the mean values of the continuous data consistent with the normal distribution was assessed using Student’s *t*-test. The Mann–Whitney U test was used to compare the continuous data inconsistent with the normal distribution. Categorized variables were compared using the chi2 test.

Moreover, we used a Cox proportional regression model to adjust for differences in baseline and lesion characteristics to reduce the effect of potential confounding factors between analyzed groups (ACS vs. CCS). The regression model included Cox proportional hazard regression (univariate and multivariate analyses) for death and MACE. The clinical outcomes were estimated using the Kaplan–Meier method and compared by the log-rank test. All *p*-values were two-sided. A value of <0.05 was considered to indicate statistical significance. Statistical Package for Social Sciences, version 23 (SPSS, IBM, Chicago, IL, USA), was used for all statistical analyses in this study.

## 3. Results

### 3.1. Clinical Characteristics

A total of 829 patients were enrolled in this study, including 185 (22.3%) with acute ACS and 644 (87.7%) with CCS, all of whom were treated with new-generation DES. Among the ACS patients, 32 (17.3%) were diagnosed with ST-segment elevation myocardial infarction (STEMI), 36 (19.5%) with non-ST-segment elevation myocardial infarction (NSTEMI), and the remaining 117 (63.2%) with unstable angina. All participants met the criteria for complex coronary artery disease. The baseline clinical and procedural characteristics are summarized in Table 1. In both the ACS and CCS groups, most patients were male, accounting for 76.2% and 74.8%, respectively. The average age was 69 years (62–75) in the ACS group and 70 years (63–76) in the CCS group.

The prevalence of heart failure was significantly higher in the CCS group, with rates of 31.5% compared to 23.2% in the ACS group (*p* = 0.03). Additionally, patients in the CCS group were significantly more likely to be obese (45.2% vs. 33.0%, *p* = 0.003) with a higher incidence of previous MI (30.8% vs. 47.4%, *p* ≤ 0.001) and PCI (50.8% vs. 62.0%, *p* = 0.006). According to the guidelines, patients in the ACS group were significantly more often treated with ticagrelor (19.5% vs. 13.5%, *p* = 0.045). The other clinical risk factors were comparable between the studied groups.

### 3.2. Procedural Characteristics

In the CCS group, lesions were prepared significantly more often with pre-dilatation (91.3% vs. 98.3%, *p* = 0.00). Additionally, the procedure was optimized more frequently with post-dilatation in the CCS group (79.6% vs. 87.1%, *p* = 0.02). The occurrence of moderate to severe calcifications (18.4% vs. 14.7%, *p* = 0.23) and multivessel disease (MVD) (45.4% vs. 42.5%, *p* = 0.49) was comparable between the groups. The length of the lesions (43 [30–56] vs. 40 [25–53], *p* = 0.023) and implanted stents (50.5 [38–66] vs. 48 [32–61], *p* = 0.024) was significantly greater in the CCS group compared to the ACS group.

The use of intravascular imaging (IVI)-guided PCI, such as intravascular ultrasound (IVUS) (4.3% vs. 4.7%, *p* = 0.85) and optical coherence tomography (OCT) (1.1% vs. 1.4%, *p* = 0.74), was low among both ACS and CCS patients. The radial approach was preferred in both groups (75.1% vs. 70.2%, *p* = 0.26, in the ACS and CCS groups, respectively). Angiographic success was achieved in almost all ACS and CCS patients (99.5% vs. 99.7%, *p* = 0.65).

The baseline procedural characteristics are summarized in Table 1 and Table 2.

### 3.3. Clinical Outcomes

**Figure 1:**
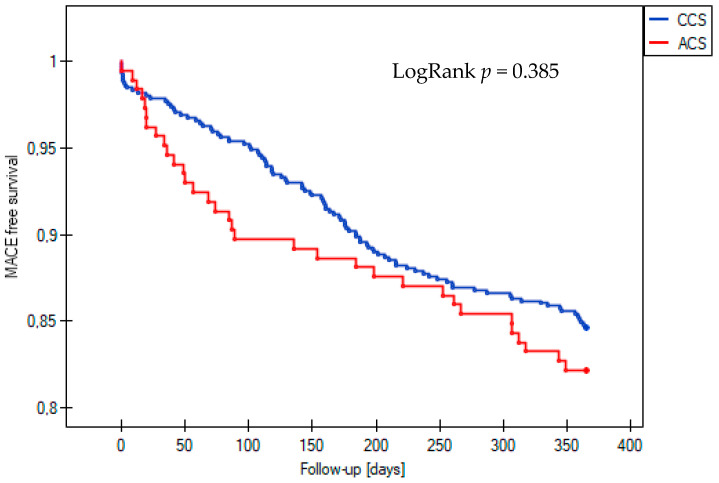
Kaplan–Meier curve for survival free from MACE during 12-month follow-up in the acute coronary syndrome (ACS) and chronic coronary syndrome (CCS) groups. MACE, major adverse cardiovascular event.

**Figure 2:**
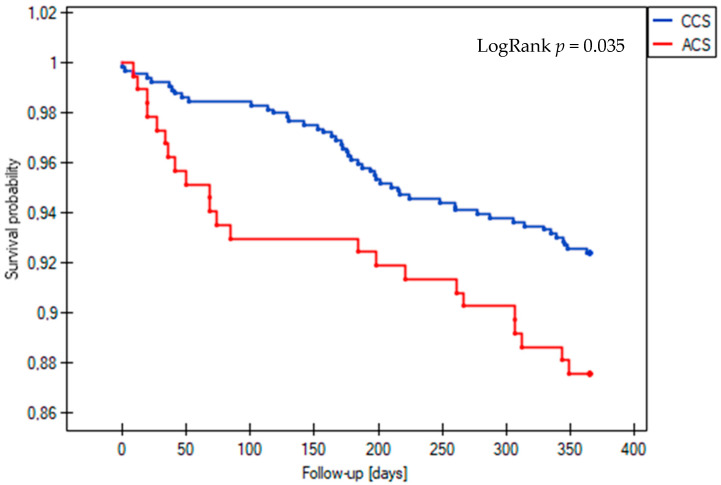
Kaplan–Meier survival curves for all-cause death during 12-month follow-up in the acute coronary syndrome (ACS) and chronic coronary syndrome (CCS) groups.

The incidence of the composite endpoint of MACE at one-year follow-up was similar between patients with acute and those with chronic coronary syndrome who underwent complex PCI, with rates of 17.8% and 15.4%, respectively (LogRank *p* = 0.385) (Figure 1). However, among the secondary endpoints, the all-cause mortality rate was significantly higher in the ACS group, at 12.4% compared to 7.6% in the CCS group (LogRank *p* = 0.035) (Figure 2). Statistical analysis revealed no significant differences in the occurrence of other clinical outcomes between the ACS and CCS groups. These included cardiovascular death (1.8% vs. 0.7%, LogRank *p* = 0.17), non-fatal MI (0.5% vs. 0.9%, LogRank *p* = 0.61), ST (0% vs. 0.5%, LogRank *p* = 0.36), TLR (3.8% vs. 5.3%, LogRank *p* = 0.42), and TVR (6.0% vs. 9.0%, LogRank *p* = 0.23).

**Figure 3:**
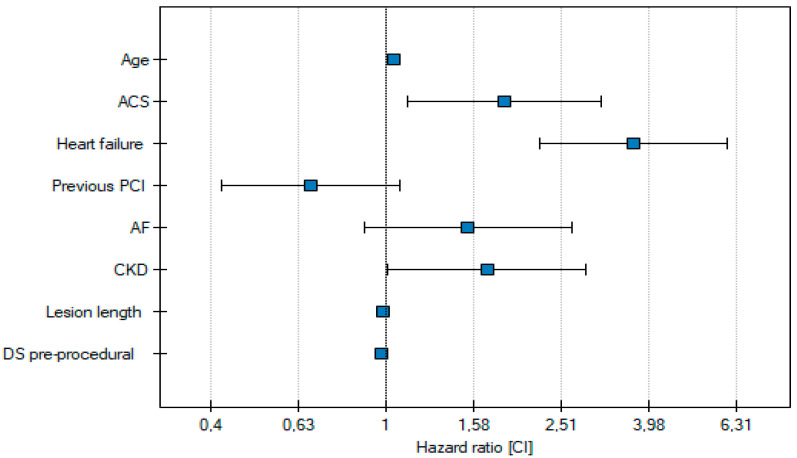
Independent risk factors for all-cause death.

Cox multivariate analysis indicated that ACS is an independent risk factor for all-cause death at one-year follow-up. Additionally, the age of the patient at the time of the procedure was identified as another independent predictor of all-cause death in individuals with complex lesions. Comorbidities such as chronic kidney disease (CKD) and heart failure, along with procedural factors including lesion length and pre-procedural diameter stenosis, are also independent predictors of death in these patients (Table 3, Figure 3).

**Figure 4:**
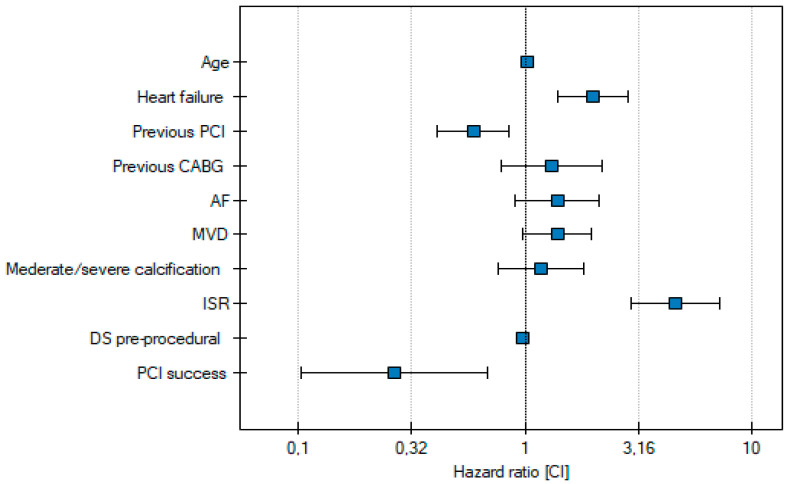
Independent risk factors for MACE.

Independent risk factors for MACE at one-year follow-up include age, heart failure, previous PCI, ISR, and pre-procedural diameter stenosis (Table 4, Figure 4).

## 4. Discussion

Our main findings indicate that the incidence of the composite endpoint of MACE at one-year follow-up is comparable between the patients with acute and chronic coronary syndrome who underwent complex PCI. However, among the secondary endpoints, the ACS group’s all-cause mortality rate is significantly higher.

Complex coronary procedures are associated with an increased risk of peri-procedural complications and long-term adverse cardiovascular events. Studies have shown a higher incidence of target lesion failure (TLF), ST, and TLR, particularly with stents longer than 40 mm. Several factors, including clinical characteristics, angiographic findings, and procedural results, can independently worsen these patients’ prognoses.

A higher prevalence of comorbidities, such as CKD and diabetes, is associated with an increased risk of reinterventions and MI, as well as the severity of coronary artery disease, including the presence of long, diffuse calcified lesions. Severe calcifications significantly contribute to suboptimal PCI outcomes, such as stent underexpansion, malposition, or significant edge dissection. Therefore, optimal preparation of the lesion, especially when it is heavily calcified, using advanced atherectomy methods is critical to achieving favorable treatment results.

ACS remains the leading cause of death worldwide. Fortunately, advancements in prevention and treatment have led to a decline in incidence. There is an increasing emphasis on intravascular IVI-guided PCI in ACS management. IVI is crucial for patients undergoing complex procedures, particularly those with moderate to severe calcifications. It allows for assessing lesion morphology and the progression of atherosclerotic lesions, directly influencing the choice of invasive treatment strategies, including selecting the appropriate atherectomy method. Additionally, optimizing procedures based on IVI findings can improve short- and long-term outcomes. However, it is essential to note that the low percentage of intracoronary imaging used in our study represents a significant limitation.

A randomized, multicenter OCTOBER trial demonstrated the superiority of OCT-guided stent implantation in bifurcation lesions compared to standard angiographic-guided implantation regarding clinical outcomes [7]. ILUMIEN IV by Ziad Ali et al. is another large-scale, multicenter, randomized trial designed to evaluate the efficacy of OCT versus angiography-guided stent implantation in patients with complex angiographic lesions and high-risk clinical characteristics in achieving larger post-PCI lumen dimensions and improving clinical outcomes [8]. The authors concluded that OCT guidance resulted in a larger minimum stent area than angiography guidance among patients undergoing PCI. There was, however, no apparent between-group difference in the percentage of patients with target-vessel failure at 2 years [9].

The RENOVATE-COMPLEX-PCI trial is another prospective, multicenter, open-label trial in South Korea. Enrolled patients with complex coronary artery lesions were randomly assigned to either intravascular imaging-guided PCI or angiography-guided PCI. The authors concluded that intravascular imaging-guided PCI was associated with a lower cumulative incidence of a composite of cardiac death, target-vessel-related MI, or clinically driven TVR than angiography-guided PCI [10].

In another study by Sung-Jin Hong et al., the authors aimed to evaluate the long-term cardiac survival benefit of IVUS versus angiography-guided DES implantation. The authors pooled the data of two randomized trials (IVUS-XPL [Impact of Intravascular Ultrasound Guidance on the Outcomes of Xience Prime Stents in Long Lesions] and ULTIMATE [Intravascular Ultrasound Guided Drug Eluting Stents Implantation in All-Comers Coronary Lesions]). They compared IVUS guidance versus angiography guidance in 2577 patients with long lesions treated with an implanted stent length of ≥28 mm. In this post hoc, pooled, patient-level analysis, the primary endpoint of cardiac death occurred in 12 patients (1.0%) in the IVUS-guided group vs. 28 patients (2.2%) in the angiography-guided group (HR: 0.43; 95% CI: 0.22–0.84; *p* = 0.011) [11].

The incidence of the composite endpoint of MACE at one-year follow-up was comparable between the patients with acute and chronic coronary syndrome who underwent complex PCI (17.8% vs. 15.4%, LogRank *p* = 0.385) (Figure 1). However, among the secondary endpoints, the all-cause mortality rate was significantly higher in the ACS group (12.4% vs. 7.6%, LogRank *p* = 0.035) (Figure 2). Statistical analysis disclosed no significant differences in the occurrence of other clinical outcomes in the ACS and CCS groups, such as cardiovascular death (1.8% vs. 0.7%, LogRank *p* = 0.17), non-fatal MI (0.5% vs. 0.9%, LogRank *p* = 0.61), stent thrombosis (0% vs. 0.5%, LogRank *p* = 0.36), and TLR (3.8% vs. 5.3%, LogRank, *p* = 0.42) or TVR (6.0% vs. 9.0%, LogRank, *p* = 0.23).

In their meta-analysis, Kevin P Liou et al. compared the clinical outcomes of patients after FFR-guided revascularization in ACS or CCS. The rates of MACE, recurrent MI, mortality, and unplanned revascularization in patients with ACS were significantly higher than in patients with CCS despite the FFR-guided revascularization strategy. Delaying revascularization does not seem as safe in ACS as in stable angina using contemporary FFR cutoffs validated in stable angina. Hence, there is a need to refine the therapeutic strategy for patients with ACS and multivessel disease to compensate for this imbalance [12].

In order to address a limitation of the current analysis, we further applied Cox proportional hazard models adjusted for clinical and angiographic factors, revealing a significantly higher hazard of all-cause death in the ACS group compared to the CCS group.

This study reveals several limitations that merit attention. First, we must note that we retrospectively compared the two groups. Additionally, there are notable baseline and procedural differences that may influence the outcomes. The very low rate of intracoronary imaging in both patient groups is particularly concerning, especially considering the complexity of these procedures. Moreover, the absence of a centralized core lab for lesion assessment means that standardization in image interpretation is lacking, potentially affecting the reliability of our findings. Because this study is retrospective, we do not have information on patient compliance. Further studies must be conducted to substantiate our observations and establish more robust conclusions.

## 5. Conclusions

The prognosis of patients with acute and chronic coronary syndrome in one-year follow-up is comparable in the context of cardiovascular events. The clinical presentation of ACS is an independent risk factor for all-cause death. To confirm the results, we still need further prospective studies to investigate the cause of death.

## Figures and Tables

**Table 1:** The baseline clinical and procedural characteristics of the overall population.

Variable	ACS (N = 185)	CCS (N = 644)	*p*-Value
Age, years	69 (62–75)	70 (63–76)	0.083
Male	141 (76.2)	482 (74.8)	0.704
Heart failure	43 (23.2)	203 (31.5)	0.030
Hypertension	159 (85.9)	567 (88.0)	0.446
Diabetes mellitus	69 (37.3)	277 (43.0)	0.165
Insulin-dependent diabetes mellitus	3 (1.6)	16 (2.5)	0.489
Previous MI	57 (30.8)	305 (47.4)	<0.001
Previous PCI	94 (50.8)	399 (62.0)	0.006
Previous CABG	10 (5.4)	63 (9.8)	0.064
Hypercholesterolemia	123 (66.5)	464 (72.6)	0.142
Smoking (current/past)	74 (40.0)	254 (39.4)	0.891
Stroke	11 (5.9)	51 (7.9)	0.368
COPD	13 (7.0)	56 (8.7)	0.469
AF	24 (13.0)	91 (14.1)	0.688
CKD (eGFR < 60 mL/min)	29 (15.8)	109 (16.9)	0.688
Obesity (BMI > 30)	61 (33.0)	291 (45.2)	0.003
EF, %	55 (49–60)	55 (45–60)	0.933
Drugs at discharge			
Prasugrel	3 (1.6)	6 (0.9)	0.425
Ticagrelor	36 (19.5)	87 (13.5)	0.045
Clopidogrel	146 (78.9)	551 (85.6)	0.030
ASA	180 (97.3)	621 (96.5)	0.564
DAPT, months	12 (12–12)	12 (6–12)	<0.001
Antithrombotic drugs	28 (15.1)	105 (16.3)	0.703
B-blocker	162 (87.6)	535 (83.1)	0.141
Calcium channel blockers	50 (27.0)	201 (31.1)	0.275
Statin	177 (95.7)	610 (94.7)	0.602
ACEI/ARB	149 (80.5)	505 (78.4)	0.533
Oral diabetes medications	49 (26.5)	216 (33.5)	0.070
Insulin	19 (10.3)	86 (13.3)	0.266
MRA	22 (11.9)	111 (17.3)	0.081
ARNI	8 (4.3)	40 (6.2)	0.333
SGLT-2 inhibitors	2 (1.1)	12 (1.9)	0.467

Values are n (%). ACEI, angiotensin-converting enzyme inhibitors; ACS, acute coronary syndrome; AF, atrial fibrillation; ARB, angiotensin receptor blocker; ARNI, angiotensin receptor/neprilysin inhibitor; CABG, coronary artery bypass grafting; CCS, chronic coronary syndrome; CKD, chronic kidney disease; COPD, chronic obstructive pulmonary disease; MI, myocardial infarction; MRA, mineralocorticoid receptor antagonist; PCI, percutaneous coronary intervention; SGLT-2, sodium–glucose cotransporter-2; significant difference = *p* < 0.05.

**Table 2:** Baseline angiographical and procedural characteristics.

Variable	ACS (N = 185)	CCS (N = 644)	*p*-Value
PCI access			
Radial	139 (75.1)	452 (70.2)	0.258
Femoral + radial	5 (2.7)	33 (5.1)	
Femoral	41 (22.2)	159 (24.7)	
Target lesion and procedure characteristics			
MVD	84 (45.4)	274 (42.6)	0.489
XVD	1 (1–2)	1 (1–2)	0.254
Bifurcation	36 (19.5)	115 (17.9)	0.619
Calcification	34 (18.4)	95 (14.7)	0.230
Rotablation	31 (16.8)	75 (11.6)	0.067
Burr size, mm	1.5 (1.5–1.5)	1.5 (1.5–1.5)	0.577
IVL	2 (1.1)	13 (2.0)	0.399
IVL balloon diameter, mm	3 (3–3)	3.5 (3–3.5)	0.090
ISR	16 (8.7)	48 (7.5)	0.591
Pre-dilatation	169 (91.4)	633 (98.3)	0.000
Pre-dilatation balloon diameter, mm	2.5 (2.5–2.5)	2.5 (2.5–2.5)	0.956
Pre-dilatation, ATM	14 (14–20)	16 (14–20)	0.595
Post-dilatation	129 (79.6)	512 (87.1)	0.017
Post-dilatation balloon diameter, mm	3.5 (3–3.5)	3.5 (3–3.5)	0.710
Post-dilatation, ATM	20 (20–20)	20 (20–20)	0.490
NC balloon	121 (93.8)	478 (93.4)	0.857
IVUS	8 (4.3)	30 (4.7)	0.848
OCT	2 (1.1)	9 (1.4)	0.740
Lesion length, mm	40 (25–53)	43 (30–56)	0.023
Reference diameter, mm	3.1 (2.8–3.2)	2.9 (2.7–3.3)	0.442
Minimal lumen diameter pre-procedural, mm	0.28 (0–0.53)	0.29 (0–0.58)	0.493
Diameter stenosis pre-procedural, %	90 (80–100)	90 (80–100)	0.364
Minimal lumen diameter past-procedural, mm	3.06 (2.75–3.18)	2.91 (2.64–3.2)	0.461
Diameter stenosis past-procedural, %	0 (0–0)	0 (0–0)	0.982
Mean stent diameter, mm	3 (2.75–3)	3 (2.5–3)	0.623
Mean stent length, mm	28 (22–33)	22 (21–38)	0.725
Mean stent, ATM	16 (16–16)	16 (16–16)	0.567
Total stent number	2 (1–2)	2 (1–2)	0.081
Total stent length, mm	48 (32–61)	50.5 (38–66)	0.024
Angiographic success	184 (99.5)	642 (99.7)	0.646
PCI success	183 (98.9)	639 (99.2)	0.690
Complications	17 (9.2)	39 (6.1)	0.285

Values are n (%) or median (IQR). ACS, acute coronary syndrome; ATM, atmosphere; CCS, chronic coronary syndrome; IVL, intravascular lithotripsy; IVUS, intravascular ultrasound; OCT, optical coherence tomography; PCI, percutaneous coronary intervention; MVD, multivessel disease; NC balloon, non-compliant balloon; XVD, X vessel disease; significant difference = *p* < 0.05.

**Table 3:** All-cause death Cox proportional hazard regression—univariate analysis and multivariate analysis.

All-Cause Death Cox Regression	Univariate Analysis		Multivariate Analysis	
	HR	*p*	HR	*p*
Age	1.07 [1.04; 1.09]	<0.001	1.04 [1.01; 1.07]	0.004
ACS	1.69 [1.03; 2.78]	0.044	1.87 [1.12; 3.11]	0.016
HF	3.96 [2.46; 6.36]	<0.001	3.68 [2.25; 6.02]	<0.001
Past PCI	0.62 [0.39; 0.99]	0.046	0.67 [0.42; 1.08]	0.100
AF	2.89 [1.75; 4.77]	<0.001	1.54 [0.89; 2.65]	0.121
CKD (eGFR < 60 mL/min)	2.33 [1.41; 3.85]	0.001	1.70 [1.00; 2.87]	0.048
Lesion length	0.98 [0.97; 0.99]	0.005	0.98 [0.97; 1.00]	0.013
Diameter stenosis pre	0.97 [0.95; 0.99]	0.015	0.98 [0.95; 1.00]	0.028

ACS, acute coronary syndrome; AF, atrial fibrillation; CKD, chronic kidney disease; HF, heart failure; PCI, percutaneous coronary intervention; significant difference = *p* < 0.05.

**Table 4:** MACE Cox proportional hazard regression—univariate analysis and multivariate analysis.

MACE Cox Regression	Univariate Analysis		Multivariate Analysis	
	HR	*p*	HR	*p*
Age	1.03 [1.01; 1.05]	0.001	1.02 [1.00; 1.04]	0.031
HF	2.00 [1.42; 2.83]	<0.001	1.98 [1.39; 2.83]	<0.001
Previous PCI	0.69 [0.49; 0.97]	0.035	0.59 [0.41; 0.85]	0.004
Previous CABG	1.72 [1.04; 2.82]	0.046	1.31 [0.78; 2.20]	0.308
AF	1.89 [1.26; 2.84]	0.002	1.39 [0.91; 2.13]	0.131
MVD	1.58 [ 1.12; 2.23]	0.008	1.38 [0.98; 1.96]	0.068
Calcification	1.59 [1.06; 2.41]	0.034	1.18 [0.76; 1.81]	0.463
ISR	3.82 [2.51; 5.80]	<0.001	4.59 [2.93; 7.21]	<0.001
Diameter stenosis pre-procedural	0.98 [0.97; 1.00]	0.033	0.98 [0.97; 1.00]	0.026
PCI success	0.14 [0.06; 0.34]	<0.001	0.26 [0.10; 0.68]	0.006

AF, atrial fibrillation; CABG, coronary artery bypass grafting; HF, heart failure; ISR, in-stent restenosis; MACE, major adverse cardiac event; MVD, multivessel disease; PCI, percutaneous coronary intervention; significant difference = *p* < 0.05.

## Data Availability

The data presented in this study are available on request from the corresponding author.
